# Amphicrine carcinoma of the stomach and intestine: a clinicopathologic and pan-cancer transcriptome analysis of a distinct entity

**DOI:** 10.1186/s12935-019-1031-7

**Published:** 2019-11-21

**Authors:** Dan Huang, Fei Ren, Shujuan Ni, Cong Tan, Weiwei Weng, Meng Zhang, Midie Xu, Lei Wang, Qinghua Xu, Weiqi Sheng

**Affiliations:** 1 Department of Pathology, Fudan University Shanghai Cancer Center, 270 Dong An Road, Shanghai, 200032 People’s Republic of China; 2 Department of Oncology, Shanghai Medical College, Fudan University, Shanghai, People’s Republic of China; 3 Institute of Pathology, Fudan University, Shanghai, People’s Republic of China; 4 CanHelp Genomics, Hangzhou, Zhejiang People’s Republic of China

**Keywords:** Amphicrine, Gastrointestinal tract, Prognosis, Pan-cancer transcriptome analysis

## Abstract

**Background and aim:**

Amphicrine carcinoma, in which endocrine and epithelial cell constituents are present within the same cell, is very rare. This study characterized the clinicopathologic and survival analysis of this tumor, further compared the genetic diversities among amphicrine carcinoma and other tumors.

**Materials and methods:**

The clinicopathologic characteristics and survival outcomes of amphicrine carcinoma in this study were analyzed. The pan-cancer transcriptome assay was utilized to compare the genetic expression profile of this entity with that of conventional adenocarcinoma or neuroendocrine tumors.

**Results:**

Ten cases (all in male patients) were identified in the stomach or intestine, with a median patient age of 62 years. There were characteristic patterns in the tumors: tubular, fusion or single-file growth of goblet- or signet ring-like cells. Four tumors were classified as low-grade and 6 as high-grade according to the histologic architecture. All cases were positive for neuroendocrine markers (synaptophysin and chromogranin A) and showed intracellular mucin in the amphicrine components. Four cases exhibited mRNA expression patterns showing transcriptional homogeneity with conventional adenocarcinomas and genetic diversity from neuroendocrine tumors. During the follow-up period, 3 patients died of disease, all of whom had high-grade tumors. Patients with high-grade amphicrine carcinoma had worse outcomes than those with low-grade tumors.

**Conclusions:**

This study confirms the morphological, immunostaining and transcriptome alterations in amphicrine carcinoma distinct from those in conventional adenocarcinomas and neuroendocrine tumors, but additional studies are warranted to determine the biological behavior and therapeutic response.

## Background

Although rare, mixed exocrine-neuroendocrine tumors have been previously described in the gastrointestinal tract [1]. Almost 30 years ago, Lewin proposed a nomenclature for dividing this unique type of tumor into three groups [2]: mixed or composite tumors, collision tumors and amphicrine tumors. Amphicrine neoplasms have been described as tumors with exocrine and neuroendocrine components in the same cell. This pattern contrasts with that in composite tumors or collision tumors, in which 2 different cellular components are admixed or juxtaposed. Since only a few studies have included amphicrine neoplasms, the use of the term “amphicrine tumor” in some studies and “amphicrine carcinoma” in others leads to great confusion in interpreting and understanding this neoplasm [3–5]. We propose “amphicrine carcinoma” as the designation to highlight its aggressive behavior.

With both neuroendocrine and exocrine differentiation, amphicrine carcinomas are significantly more likely to have unique features in histopathology. However, the descriptions of amphicrine carcinomas in terms of morphology and immunophenotype have, to date, been limited in previous reports. In the clinic, the dual nature of these tumors is still largely unrecognized, and there is no unified concept of how to treat patients with amphicrine carcinoma.

To gain a better understanding of the biological properties of amphicrine carcinoma, it is essential to study the genetic profiles of these tumors. Recently, close genetic relations have been revealed in mixed adenoneuroendocrine carcinomas (MANECs) and adenocarcinomas [6]. Another next-generation sequencing study focusing on somatic mutations and driver genes also suggested a monoclonal origin for different components of MANEC [7]. However, a comparison of molecular characteristics, especially mRNA levels, between amphicrine carcinomas and adenocarcinomas or neuroendocrine tumors is needed.

Our study, the largest case series to date, aimed to explore the clinicopathological features of amphicrine carcinoma in the stomach and intestine via hierarchical clustering analysis using a pan-cancer transcriptome panel in an effort to more appropriately define the specific morphology, clinical behavior and genetic differences of this neoplasm from other neoplasms.

## Materials and methods

### Case selection

This study was performed in accordance with local ethical and legal requirements after approval by the Ethics Committee of Fudan University Shanghai Cancer Center (FUSCC), and written informed consent was obtained from all participants or their appropriate surrogates. A total of ten cases of amphicrine carcinoma of the stomach or intestine were retrieved from the consultation and surgical pathology files of the Department of Pathology at FUSCC between 2009 and 2017. Available medical records, including imaging study reports, were reviewed to obtain clinical data such as age, sex, presenting symptoms, endoscopic descriptions, treatment, and outcome; for the consultation cases, contributing physicians were contacted.

### Pathologic features

Available gross images, descriptions and histologic sections were reviewed by three pathologists (authors: DH, FR and SJN) to confirm the diagnosis and further characterize the histological findings. The pathologic criteria for diagnosis of amphicrine carcinoma requires exocrine and neuroendocrine features in the same neoplastic cell, which shows a divergent immunophenotype [1]. According to histologic features, our cases were divided into low, intermediate and high grades using the grading system recommended by Yozu [8]. This methodology is used to grade appendiceal goblet cell carcinomas by assessing the proportion of the tumor with tubular or clustered growth. Tumors with > 75% tubular or clustered growth were classified as having a low-grade pattern, which is characterized by small and cohesive clusters of goblet cells with or without lumina. The cells in these clusters had low to at most moderate cytologic atypia and infrequent mitoses, sometimes with peripheral localization of nuclei. Tumors with 50% to 75% tubular growth were classified as having an intermediate-grade pattern, and tumors with < 50% tubular growth were classified as having a high-grade pattern. These growth patterns deviated from the low-grade pattern and showed several forms, including single-file or sheet-like growth of signet ring-like cells. Clusters of tumor cells, especially in mucin-poor areas, had increased cytologic atypia and increased mitotic activity.

### Immunohistochemistry

Immunohistochemistry for pankeratin (clone AE1/3, 1:150, Dako), CgA (clone LK2H101 + PHE5, Roche), Syn (clone MRQ-40, 1:400, Roche), CD56 (clone 123C3, 1:80, Dako) and Ki67 (clone 30-9, Roche) were performed using a Ventana BenchMark XT Automated Staining System. Alcian blue staining was performed to evaluate mucin content using an Alcian blue staining kit (BASO) following the manufacturer’s instructions.

### Pan-cancer transcriptome assay

The genetic data generated for amphicrine carcinomas were compared with data from a set of neuroendocrine tumors and gastric adenocarcinomas, which were genetically analyzed by the same 90-gene real-time PCR assay [9]. In brief, manual macrodissection of tumor-rich areas from unstained slides of formalin-fixed, paraffin-embedded tissue was performed under microscopy with guidance from hematoxylin and eosin staining. Total RNA was isolated from formalin-fixed, paraffin-embedded (FFPE) tissue sections using an FFPE Total RNA Isolation Kit (Canhelp Genomics, Hangzhou, China). The concentration and purity of total RNA were determined by spectrometry according to the manufacturer’s instructions. Next, cDNA was generated from isolated total RNA using a high-capacity cDNA Reverse Transcription Kit with RNase Inhibitor (Applied Biosystems, Foster City, CA, United States). The A260/A280 of total RNA isolated from tissue sections from amphicrine carcinoma, NET and gastric cancer patients was 1.89–2.00. The expression level of each of the 90 genes was measured in an Applied Biosystems 7500 Real-Time PCR system using TaqMan Gene Expression Assays (Applied Biosystems). Normalized gene expression intensities were shifted to set the mean to 0 and rescaled to set the STD to 1 to enhance the expression differences. The average linkage hierarchical clustering method was performed, where the metric of similarity was the Pearson correlation between every pair of samples. In addition, relative mRNA expression intensities were triple detected for each specimen of all samples, including amphicrine carcinoma (AC), neuroendocrine tumor (NET) and stomach adenocarcinoma (STAD) samples.

Biological network analysis and KEGG (Kyoto Encyclopedia of Genes and Genomes) pathway analysis were performed using NetworkAnalyst software (version 3.0) (Cite 10.1093/nar/gkz240). Protein–protein interactions have been retrieved from IMEx Interactome Database (Cite 10.1093/nar/gks1147). A minimum network was generated by keeping seed proteins as well as minimum essential non-seed proteins to study the fundamental interactions.

### Statistical analysis

Statistical analyses to compare clinicopathologic characteristics and overall survival were performed using SPSS (version 20.0, IBM). Means and ranges are used to describe quantitative variables. Overall survival curves were generated by the Kaplan–Meier method, and the log-rank test was used in difference analyses. A *P* value of < 0.05 was considered statistically significant.

## Results

### Clinical features

The clinical features and staging parameters for our ten amphicrine cancer cases are summarized in Table 1. All patients were male, with a mean age of 63 years (ranging from 56 to 69). The presenting symptoms in seven patients included upper abdominal pain, hematochezia and hematemesis. The eight gastric neoplasms were located throughout the stomach, and the antrum was the most commonly involved region. Another two cases in the intestine arose from the rectum. According to the histologic evaluation of tumor grades, most low-grade tumors were in an early T stage (T1 or T2). No patient had nodal or distant metastasis at presentation; however, one patient with stage IIIA disease had one lymph node involved. Lymphovascular invasion was present in only that patient (25%), and perineural invasion was not observed in any low-grade sample. In contrast, the high-grade group and mixed group had a high proportion of late-stage disease. The percentage of patients with lymph node involvement was increased (75%), as did the percentage of patients with synchronous distant metastasis (33%). Perineural invasion was observed in 2 high-grade tumors, and lymphovascular invasion was present in 3 cases.

**Table 1:** Clinical features and follow-ups

Case	Gender	Age (years)	Specimen type	Position	Gross	Size (cm)	T	N	M	Stage^a^	Pl	LVI	Treatment	Outcomes
1	M	61	Resection	Gastric body	Ulcerative	5.5	T4	1/17	0	IIIA	N	P	Surgery + chemotherapy	NED, 63 months
2	M	61	Biopsy	Gastric antrum	NA	NA	T1	NA	NA	NA	NA	NA	NA	NED, 12 months
3	M	58	Resection	Gastric antrum	Ulcerative	NA	T2	0/18	0	IB	N	N	Surgery	NED, 10 months
4	M	63	Resection	Gastric antrum	Ulcerative	3.0	T1	0/27	0	IA	N	N	Surgery	NED, 10 months
5	M	56	Resection	Gastric body	Ulcerative	2.5	T4	6/15	0	IIIA	N	P	Surgery	DOD, 42 months
6	M	68	Resection	Gastric antrum	Ulcerative	4.5	T4	28/47	0	IIIC	P	P	Surgery + chemotherapy	NED, 12 months
7	M	60	Resection	Gastric cardia	Ulcerative	2	T4	1/21	0	IIIA	P	N	Surgery	DOD, 11 months
8	M	65	Biopsy	Rectum	Fungating	5	NA	NA	1^b^	NA	NA	NA	Palliative radiotherapy and chemotherapy	DOD, 12 months, liver and lung metastasis
9	M	67	Resection	Gastric cardia	Ulcerative	3.5	T3	0/16	0	NA	N	P	Surgery + chemotherapy	NED, 6 months
10	M	69	Biopsy	Rectum	Fungating	3.0	cT4	NA	1^b^	NA	NA	NA	Chemotherapy	AWD, 33 months, liver metastasis

*M* Male, *PI* perineural invasion, *LVI* lymphovascular invasion, *P* positive, *N* negative, *NA* not available, *AWD* alive with disease, *DOD* dead of disease, *NED* no evidence of disease

^a^Stage is according to the AJCC 8th edition gastric cancer staging system

^b^With synchronous liver metastases

### Pathologic findings

**Fig. 1:**
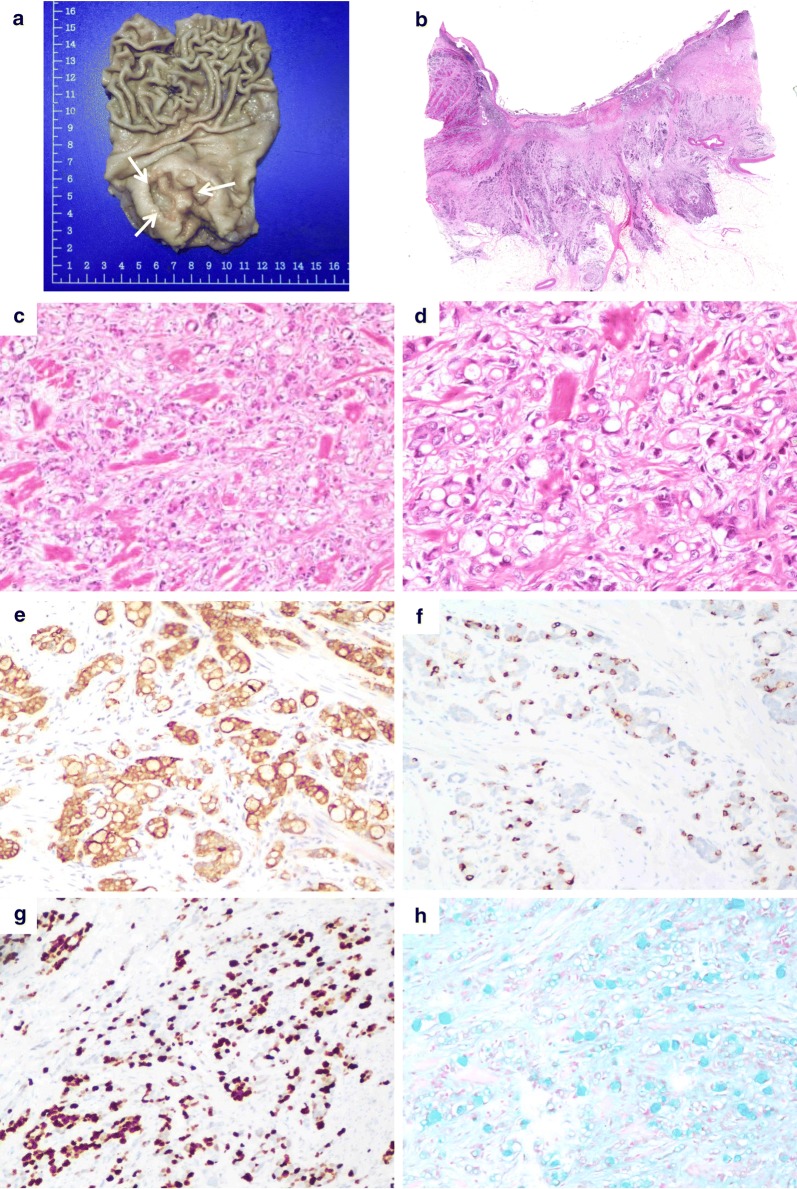
Amphicrine carcinoma with a high-grade pattern (case 6). **a** Ulcerative mass in the gastric angle, gross appearance. **b** Destructive infiltration with extension into the subserosal tissue. **c** Fusion and disorganized growth of amphicrine carcinoma cells, ×400. **d** Infiltrating signet ring-like cells with nuclei compressed to the periphery by abundant intracellular mucin, ×400. **e** Positive staining of synaptophysin, ×200. **f** Focal positive staining of chromogranin A, ×200. **g** Immunostaining of Ki67, ×200. **h** Staining of intracellular and extracellular mucin by Alcian blue, ×200

Grossly, the tumor sizes ranged from 2 to 5 cm (mean, 3.6 cm) in the maximum dimension. In the 9 cases for which endoscopic or gross information was available, the ulcerative nature of the tumor was described in seven (Fig. 1a). The remaining two tumors were documented as fungating lesions. After assessment of tubular and clustered components, 4 tumors were categorized as low-grade, 6 as high-grade, and none as intermediate-grade.

**Fig. 2:**
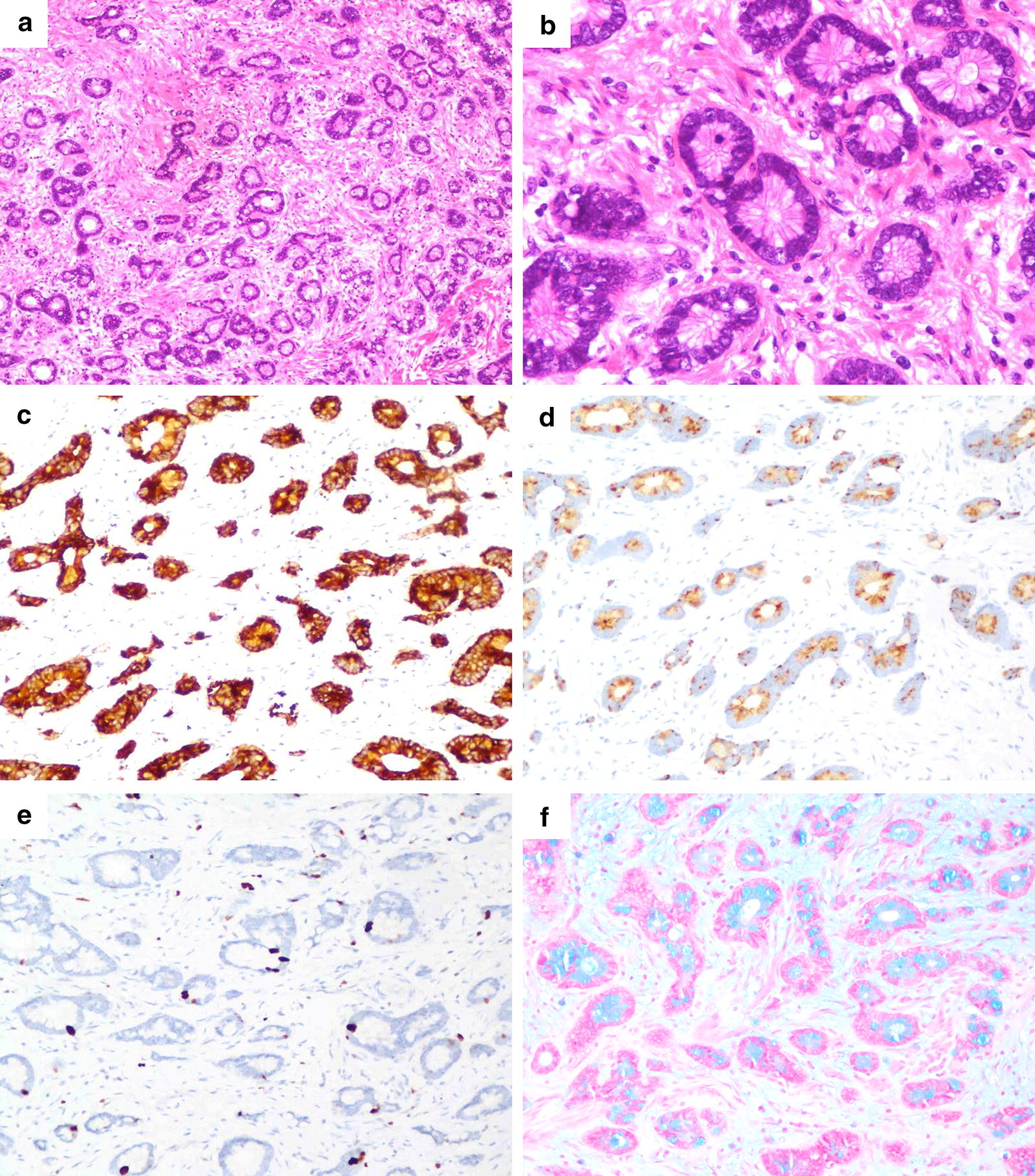
Amphicrine carcinoma with a low-grade pattern (case 4). **a** Small tumor clusters with lumens and peripheral placement of nuclei, ×100. **b** Well-formed tubules comprising goblet-like mucinous cells, ×400. **c** Diffuse positive staining for synaptophysin, ×200. **d** Paranuclear dot-like immunostaining of chromogranin A, ×200. **e** Immunostaining of Ki67, ×200. **f** Intracellular positivity for Alcian blue staining, ×200

**Fig. 3:**
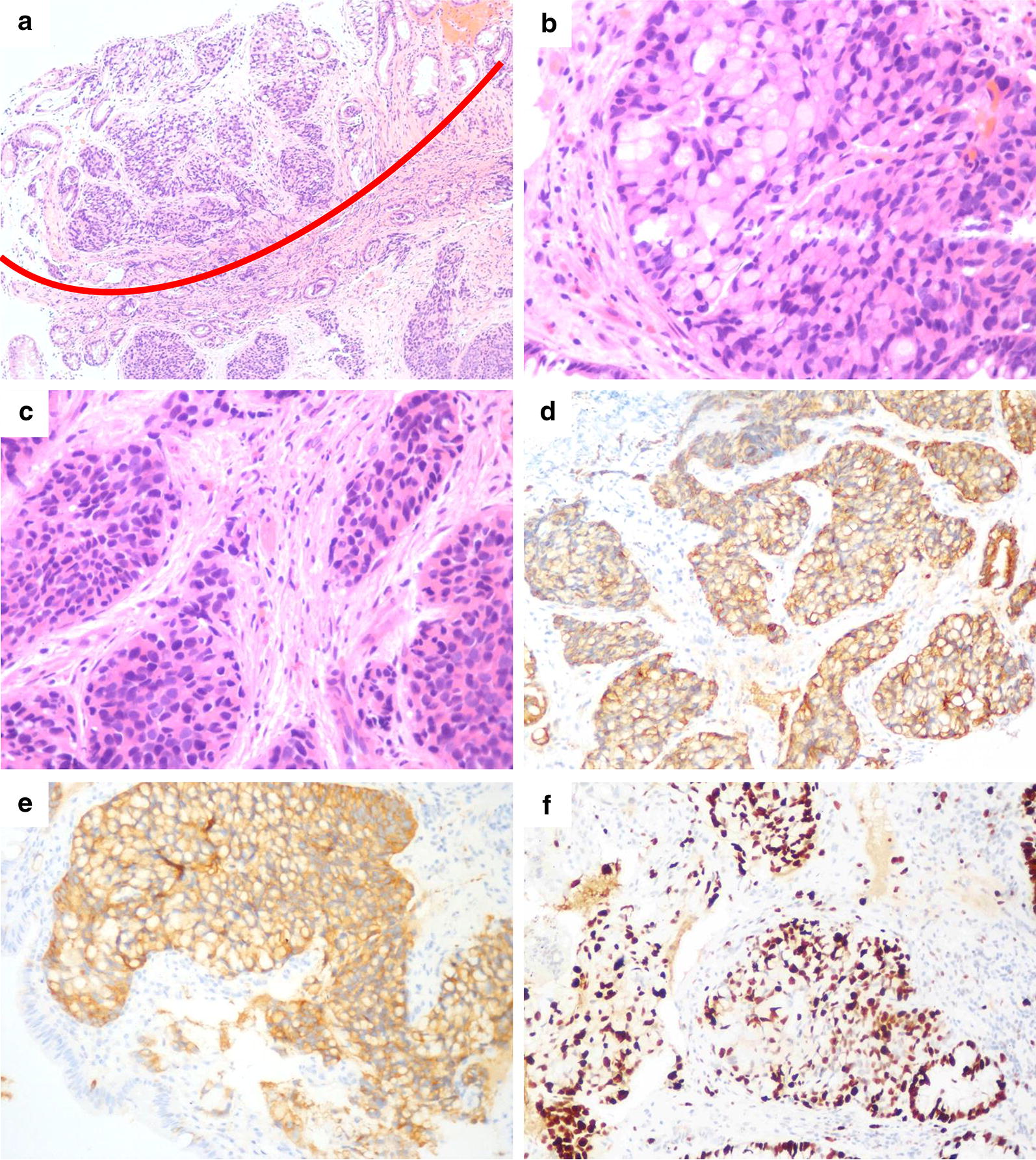
Mixed amphicrine-neuroendocrine carcinoma (case 10). **a** Admixture of amphicrine carcinoma (upper) and neuroendocrine carcinoma (bottom), in which each constituent was present in equivalent amounts, ×100. **b** Clusters of disorganized goblet cells in the high-grade amphicrine carcinoma area, ×400. **c** Neuroendocrine carcinoma component with a traditional small cell carcinoma appearance, ×400. **d** Positive staining of AE1/AE3 in the amphicrine component, ×200. **e** Positive staining of synaptophysin in the amphicrine component, ×200. **f** Immunostaining of Ki67 in the amphicrine component, ×200

The histology was somewhat complicated but showed three components consisting of three types of neoplasms: (1) low-grade amphicrine carcinoma (may mix with other components but less than 30% of tumor cell population); (2) high-grade amphicrine carcinoma (may mix with other components but less than 30% of tumor cell population); and (3) mixed amphicrine-neuroendocrine carcinoma (amphicrine carcinoma and other carcinoma, each of which according for more than 30% of tumor cell population). After assessment of the components and grades, 4 tumors were in the low-grade group, 4 were in the high-grade group, and 2 were in the mixed group (Table 2). In the low-grade group, one patient had another minor conventional adenocarcinoma component comprising 5% of the tumor. By definition, the low-grade group included tumors with up to 25% high-grade components, but none of the included tumors showed combination with any high-grade components. The most common histologic architectures in the low-grade category were tubular growth with intracellular mucin and peripheral placement of nuclei (Fig. 2), which resembled goblet cell carcinoid/carcinoma in the appendix. None of the cases in this group had single-file infiltration by signet ring-like cells. In 2 cases with an extracellular mucin pool, the tumor clusters maintained their cohesive, uniform appearance, and resembled disrupted intestinal crypts. In the high-grade group, one case presented one area of conventional adenocarcinoma (5%) and another area of low-grade amphicrine cancer characterized by tubular structures lined by goblet-like cells (45%). Another high-grade case had a low-grade component of 5%. Some nontubular growths were common among the high-grade tumors, which deviated from the architecture in the low-grade group. The most frequent forms were fusion of goblet cell clusters and disorganized growth by signet ring-like cells. Single files of goblet cells or signet ring-like cells were also fairly common (Fig. 1). Less common patterns included mucin-poor areas composed of tumor nests with high cytologic grade that resembled conventional adenocarcinoma and tumor clusters floating in mucin formed by signet ring-like cells with nuclear atypia and mitotic frequency. There were 2 cases of mixed amphicrine-neuroendocrine carcinoma that had a high-grade amphicrine component intermixed with areas of conventional neuroendocrine carcinoma (NEC), comprising 50% and 60% of the tumor (Fig. 3). Similar to the growth patterns in the high-grade group, the amphicrine components were aggregates and fusions of goblet cells forming complex disordered structures, which differentiated these areas from typical NEC areas in histologic architecture.

**Table 2:** Histologic features of amphicrine cancer

Case	Group	Grade^a^	Other components	Histologic architecture	Grade percent		Conspicuous nucleoli	Mitosis (per 10 HPF)	Mucin		Genetic analysis
					Low	High			IC	EC	
1	Low	Low	Adenocarcinoma 5%	Tubular and sheet-like growth	95	0	–	2	+	+	
2	Low	Low	–	Tubular growth	100	0	–	1	+	+	Yes
3	Low	Low	–	Tubular and sheet-like growth	100	0	–	2	+	−	Yes
4	Low	Low	–	Tubular and sheet-like growth	100	0	–	6	+	−	
5	High	High	–	Thick trabecular and fusion growth	0	100	+	24	+	−	
6	High	High	–	Single files, fusion and disorganized growth	0	100	–	11	+	+	Yes
7	High	High	Adenocarcinoma 5%	Single files and tubules growth	45	50	+	32	+	+	Yes^a^
8	High	High	–	Fusion and disorganized growth	5	95	–	20	+	−	
9	Mixed	High	NEC 60%	Fusion and disorganized growth	0	40	–	12	+	−	
10	Mixed	High	NEC 50%	Fusion and disorganized growth	0	50		20	+	−	

*NEC* Neuroendocrine carcinoma, *IC* intracellular, *EC* extracellular, *HPF* high power fields

^a^High-grade Amphicrine area was selected to perform genetic assay

The immunohistochemical staining results for all cases is summarized in Table 3. All tumor components were positive for cytokeratin (AE1/3). Amphicrine components in all cases had at least focal or patchy positivity for synaptophysin and chromogranin A. CD56 staining, if performed, also showed cytoplasmic positivity in most tumors. Those neuroendocrine markers did not differ between the low-grade and high-grade groups, only indicating the amphicrine differentiation of tumor cells. Mucin was visualized by Alcian blue staining, identifying the goblet cells and signet ring-like cells with true intracellular mucin in amphicrine components. The range of the Ki67 index was 5–40% in low-grade tumors and 20–70% in high-grade tumors, suggesting a difference in cell proliferation between the two groups.

**Table 3:** Immunohistochemical and special staining results

Case	AE1/3	CgA^a^	Syn^a^	CD56^a^	AB^a^	Ki-67^a^
1	+	+	+	–	+	5%
2	+	+	+	/	+	/
3	+	+	+	–	+	35%
4	+	+	+	+	+(P)	40%
5	+	+	+	/	+(P)	20%
6	+	+(P)	+	/	+	70%
7	+	+	+	+(F)	+	30%
8	+	+	+	/	+(P)	60%
9	+	+(P)	+	+(P)	+(P)	60%
10	+	+(F)	+	+	+(P)	70%

*AB* Alcian blue, *CgA* chromogranin A, *F* focal (< 10% labeling), *P* patchy (11 to 49% labeling), *Syn* synaptophysin

^a^In amphicrine component; / indicates not performed

### Pan-cancer transcriptome analyses

**Fig. 4:**
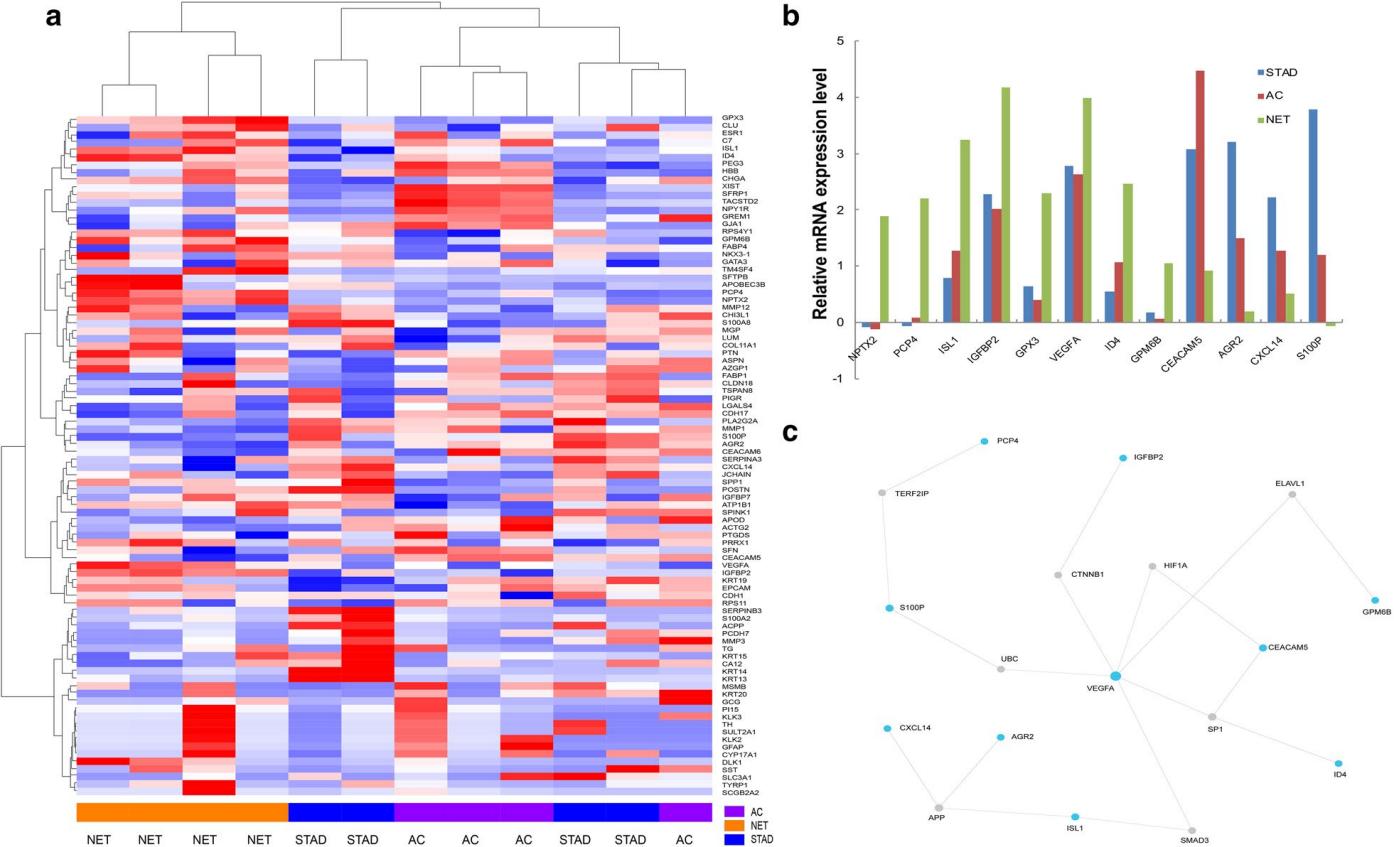
Expression profiling and survival of patients with amphicrine carcinoma. **a** Hierarchical clustering analyses of amphicrine carcinoma samples. The colored pixels indicate the magnitude of expression of any gene, where the shades of red and blue represent overexpression and underexpression, respectively, relative to the mean expression level of each gene. Heatmap representation of the normalized mRNA levels of 90 genes (rows) in the tumor samples (columns), including amphicrine carcinoma (AC) in purple, neuroendocrine tumor (NET) in orange, and gastric adenocarcinoma (STAD) in blue. All AC patients were clustered into the STAD type, differentiated from the NET group. **b** Relative mRNA expression intensity for 12 genes. A total of 12 genes were selected for profiling based on their significant differences among the 3 groups. **c** Minimum protein–protein interaction network of the 12 genes. Blue nodes indicate the proteins involved in the 12-gene set, whereas grey nodes represent proteins absent in the 12-gene set. The size of the node is proportional to the degree of connections

A previous study established a 90-gene expression signature to accurately classify a broad spectrum of tumor types [10]. In our research, the genetic data generated for amphicrine carcinomas, 4 cases with available blocks (Table 2), were compared with data from a set of four neuroendocrine tumors and four gastric adenocarcinomas. The average linkage hierarchical clustering method was performed, where the metric of similarity was the Pearson correlation between the 90-gene expression profiles of the samples (Fig. 4a). The heatmap representation of mRNA expression shows that all amphicrine carcinomas and conventional adenocarcinomas were clustered together. Relatedness of clusters was identified between amphicrine carcinomas and NETs. The relative expression intensity of the 12 genes with the greatest variation was used to differentiate related groups in these samples (Fig. 4b). The expression levels of the NPTX2, PCP4, ISL1, IGFBP2, GPX3, VEGFA, ID4 and GPM6B genes were synchronously decreased in the AC and STAD groups compared with their expression levels in NET entities. High levels of CEACAM5, AGR2, CXCL14 and S100P gene expression were simultaneously observed in the AC and STAD groups, while the NET group had significantly lower expression levels of those genes. In the further analysis of the minimum protein–protein network, 12 genes were generated as the seeds, and 10 of them are centered on VEGFA node (Fig. 4c). Next, enrichment analysis of genes was performed in the network. It revealed that the most significant KEGG pathways were signaling pathways regulating pluripotency of stem cells, TGF-β signaling pathway, pathways in cancer, etc. (Additional file 1: Table S1). Upon inspection, it was evident that amphicrine carcinoma exhibited transcriptional homogeneity with conventional adenocarcinoma and genetic diversity from NET. Moreover, the gene profiles in the low-grade and high-grade groups were indistinguishable in our limited cohort, exhibiting similar mRNA expression levels in low-grade (cases 2 and 3) and high-grade (cases 6 and 7) tumors.

### Treatment and survival outcomes

**Fig. 5:**
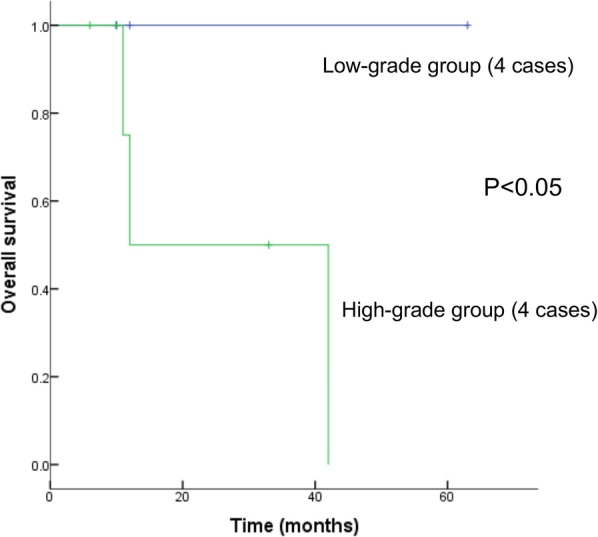
Kaplan–Meier survival curves for the cohort based on tumor grade, comparing the low-grade group with the high-grade group

Regarding treatment, seven patients underwent complete resection, and three of these received chemotherapy after surgery. Two patients received adjuvant chemotherapy or chemoradiotherapy after biopsy. One patient with early-stage disease did not receive any treatment. Survival information was available for all patients (Table 1), with a follow-up period ranging from 6 to 63 months. A total of 3 patients died of disease—at 11, 12 and 42 months, and all had tumors with a high-grade pattern. Seven patients were alive after a follow-up period ranging from 6 to 63 months. Of these patients, 6 had experienced no recurrence or metastasis after treatment; 4 of these patients had low-grade tumors. The survival rate was 100% in the low-grade group, compared to 50% in the high-grade group (P < 0.05). Kaplan–Meier survival curves showed a dramatic difference in overall survival between the low-grade and high-grade groups (Fig. 5), suggesting that high-grade morphology is associated with poorer prognosis.

## Discussion

The coexistence of endocrine and exocrine secretory products within single cells was first suggested by Feyrter in 1938 [11]. Later, Ratzenhofer advocated the term “amphicrine” for cells synchronously displaying exocrine and endocrine differentiation [12]. In 1987, Lewin proposed a simple nomenclature for dividing mixed exocrine-neuroendocrine tumors into three groups [2]: mixed or composite tumors, collision tumors and amphicrine tumors. Then, Lewin and Appelman revised the nomenclature into five categories [13], including (1) carcinomas with interspersed NE cells, (2) composite glandular-endocrine carcinomas, (3) collision tumors, (4) amphicrine tumors, and (5) combinations of the first 4. Since that time, investigators have further subdivided this unique tumor into additional categories [1, 14]. However, the terminology and classification of amphicrine tumors are still controversial. The terms that have been used to describe amphicrine neoplasms in the GI tract include goblet cell carcinoid (GCC) [15], goblet cell carcinoma [16], amphicrine tumor [3, 4] and amphicrine carcinoma [5, 17]. The current (2010) World Health Organization designated only appendiceal amphicrine neoplasms as GCCs, classified as a special subtype both in neuroendocrine neoplasms (NENs) and in adenocarcinomas [18]. Further studies revealed the aggressive clinical behavior of GCCs [15, 19], which supported the change from “goblet cell carcinoid” to “goblet cell carcinoma” [8, 16]. This terminology facilitates the staging and clinical treatment of these tumors as adenocarcinomas, not as NENs, with the AJCC recommendations [20]. For non-appendiceal GI tract tumors, different terms produce additional confusion, even potentially misleading diagnosis and treatment. Some investigators used the term “extra-appendiceal GCC”, which should, in the words used, carefully differentiate these tumors from extra-appendiceal metastasis of primary appendiceal GCC [21]. Since the word “amphicrine” provides an appropriate description of hybrid epithelial-neuroendocrine neoplasms, we advocate using the term “amphicrine carcinoma”, a term that accounts for these unique lesions of intermediate malignancy, for these cases.

In this study, we analyzed 10 cases, including 8 lesions arising in the stomach and 2 in the intestine. All of our patients were male, with a median age of 62 years (range 56–68 years). The predominant locations were the antrum in the stomach and the rectum in the intestine. Most patients presented with advanced-stage disease and lymph node metastasis. Similarly, involvement of lymphoid tissue was found in the previous case reports [4, 22]. Morphologically, these cases could be divided into pure amphicrine carcinoma and amphicrine carcinoma mixed with neuroendocrine carcinoma (NEC) or adenocarcinoma. The latter mixed pattern has also been observed in the appendix (adenocarcinoma ex-GCC), which is associated with a worse outcome than pure GCC [9, 23]. In terms of morphologic criteria, amphicrine carcinomas with bidifferentiation indicated by staining were difficult to classify with the current grading system based on common adenocarcinoma. For appendiceal GCC tumors, several grading systems have been proposed to classify patients into prognostically relevant groups [24, 25]. We followed Yozu’s grading system [8], assessing the proportion of the tumor exhibiting tubular or clustered growth, to subgroup our cases into 6 high-grade cases and 4 low-grade cases. To date, limited survival information has been available in studies of amphicrine carcinomas of the stomach and intestine. In a literature review, Nugent et al. [26] suggested that these carcinomas behave less aggressively, with a better outcome, than other tumors in these locations. In contrast, previous studies in appendiceal GCC indicated that tumor grade is the major influence on clinical presentation and prognosis [8, 27]. Our results supported the latter view that the histologic grade is closely correlated with overall survival. As shown in case 3, this patient had a pure low-grade lesion in stage IIIA (T4N1), but lived for 63 months. Thus, histologic grading might be exerted an effect on tumor survival in amphicrine carcinomas.

The morphologic features of the amphicrine component resemble the previously described clinicopathologic findings in case reports of stomach neoplasms. Young et al. [28] reported a case of amphicrine carcinoma of the stomach that was arranged in a classic carcinoid pattern of solid nests and tubules and confirmed to exhibit biphasic differentiation by electron microscopy. Fujiyoshi et al. [29] also reported two composite carcinomas of the stomach with a GCC component formed by goblet carcinoid cells in tubules and rosette-like structures. In our cases, the histological characteristics distinguished different grades. All low-grade amphicrine cancers had the morphologic appearance of tubular growth. Some high-grade cases showed single-file cell infiltration, which represents a pattern of cancer cell spread. In addition to their distinct architectural patterns, low-grade tumors were more likely to show a lower cytologic grade, lower N/C ratio and less mitosis (average of 2.75/10 HPF) than high-grade tumors with more malignant aspects. However, there was no difference in the presence of intracellular or extracellular mucin. All cases exhibited expression of at least one neuroendocrine marker (Syn, CgA or CD56), and no differences were found in the levels of these markers among different grades. Alcian blue staining, which was available in all examined cases, was helpful in identifying exocrine function. Notably, an increase in the mitosis rate and Ki67 proliferation index was readily observed in high-grade cases, with an average index of 22% in the low-grade group and 52% in the high-grade group. However, the role of the Ki67 index was quite different in studies of appendiceal GCC; thus, the prognostic value of the proliferation rate is still controversial [24, 30]. Although in limited cases in our study, Ki67 index seems not a predictive marker for prognosis in amphicrine carcinomas, which needs further studies.

The frequency of amphicrine carcinoma of the stomach and intestine may be underestimated in current diagnostic practice. Indeed, the amphicrine component may be misinterpreted as a signet ring cell formation of an adenocarcinoma if expression of neuroendocrine markers is not found by immunohistochemistry. Notably, a relatively high frequency of neuroendocrine positivity was found in previous studies of signet ring cell carcinomas; these studies reported immunostaining for neuroendocrine markers in approximately 40% of cases [31]. One study limited to staining for chromogranin A, a sensitive marker of neuroendocrine differentiation, also demonstrated focal or diffuse immunopositivity in 37.3% of gastric signet ring cell carcinomas, including 6% with staining in more than half of neoplastic cells [32]. In these previously cited studies, cells with neuroendocrine staining in signet ring carcinoma appear to represent amphicrine differentiation, demonstrating the distinct cytologic and architectural features of these tumors from those of composite tumors of mixed signet ring cell carcinoma and NEC. In our study, amphicrine carcinomas with other adenocarcinoma or NEC components were found in 4 of 10 cases, showing a high frequency of mixed growth patterns. Thus, pure amphicrine carcinoma is unusual, but the amphicrine component in mixed form is not as rare due to underdiagnosis and neglect in reporting.

Amphicrine carcinoma is a unique entity with distinct biological and histological features. However, its genetic background and molecular relationship to adenocarcinoma/NEC is largely unknown. Previous studies revealed that different components of mixed adenoneuroendocrine carcinomas have similar mutation profiles, suggesting a developmental relationship between neuroendocrine carcinoma and conventional adenocarcinoma [6, 7, 33]. Recently, a study of appendiceal goblet cell carcinoids revealed the mutational distinction between goblet cell carcinoids and neuroendocrine neoplasms/adenocarcinomas [34]. These investigations appear to be quite limited and controversial. To comprehensively analyze the transcriptomic profile of amphicrine carcinoma, we performed pan-cancer transcriptome analyses in 12 patients, including patients with amphicrine cancer, adenocarcinoma and NEC. This gene expression signature was established in a comprehensive database integrating microarray- and sequencing-based gene expression profiles. Previous studies had demonstrated the excellent performance of the 90-gene expression signature for identification of tumor origin [10, 35]. After hierarchical clustering of the gene expression magnitudes, the pan-cancer panel reflected the similarity between the mRNA expression profile in amphicrine carcinoma and traditional adenocarcinoma, with no relationship between the amphicrine carcinoma and NET profiles. In the minimum protein–protein network and enrichment analysis, genes from amphicrine carcinoma were mostly related to VEGFA node and pathways in cancer. These findings provide additional insight into the nature of amphicrine carcinoma. Interestingly, the possibility that amphicrine carcinomas are genetically related to adenocarcinomas raises the question of whether adenocarcinoma-targeted treatments would display a response in amphicrine entities with molecular alterations.

## Conclusion

In summary, amphicrine carcinoma is a distinct clinicopathologic entity in the stomach and intestine. The survival outcome of this malignancy is related to the histologic grade, and its rarity might result in significant underdiagnosis, especially in mixed form. The pan-cancer transcriptome analysis revealed that amphicrine carcinoma is genetically linked to adenocarcinoma instead of to neuroendocrine tumors. Further studies are warranted to determine whether these tumors may be susceptible to adenocarcinoma-targeted treatments.

## Supplementary information

**Additional file 1: Table S1.** Enriched KEGG pathways of 18 genes within network (p value <0.05).

## Data Availability

Not applicable.

## Abbreviations

MANEC: mixed adenoneuroendocrine carcinomas
AC: amphicrine carcinoma
NET: neuroendocrine tumor
STAD: stomach adenocarcinoma
GCC: goblet cell carcinoid
NEN: neuroendocrine neoplasm
NEC: neuroendocrine carcinoma
